# Performance of uncertainty-based active learning for efficient approximation of black-box functions in materials science

**DOI:** 10.1038/s41598-024-76800-4

**Published:** 2024-11-06

**Authors:** Ai Koizumi, Guillaume Deffrennes, Kei Terayama, Ryo Tamura

**Affiliations:** 1 Center for Basic Research on Materials, National Institute for Materials Science, 1-1, Namiki, Tsukuba, Ibaraki 305-0044 Japan; 2 Univ. Grenoble Alpes, CNRS, Grenoble INP, SIMaP, F-38000 Grenoble, France; 3 Graduate School of Medical Life Science, Yokohama City University, 1-7-29, Suehiro-cho, Tsurumi-ku, Yokohama, Kanagawa 230-0045 Japan; 4 MDX Research Center for Element Strategy, Tokyo Institute of Technology, 4259, Nagatsuta-cho, Midori-ku, Yokohama, Kanagawa 226-8501 Japan; 5 RIKEN Center for Advanced Intelligence Project, 1-4-1, Nihonbashi, Chuo-ku, Tokyo 103-0027 Japan; 6 Graduate School of Frontier Sciences, The University of Tokyo, 5-1-5, Kashiwa-no-ha, Kashiwa, Chiba 277-8561 Japan

**Keywords:** Active learning, Alloy, Semiconductor, Polymer, Molecule, PHYSBO, Materials science, Techniques and instrumentation, Design, synthesis and processing

## Abstract

Obtaining a fine approximation of a black-box function is important for understanding and evaluating innovative materials. Active learning aims to improve the approximation of black-box functions with fewer training data. In this study, we investigate whether active learning based on uncertainty sampling enables the efficient approximation of black-box functions in regression tasks using various material databases. In cases where the inputs are provided uniformly and defined in a relatively low-dimensional space, the liquidus surfaces of the ternary systems are the focus. The results show that uncertainty-based active learning can produce a better black-box function with higher prediction accuracy than that by random sampling. Furthermore, in cases in which the inputs are distributed discretely and unbalanced in a high-dimensional feature space, datasets extracted from materials databases for inorganic materials, small molecules, and polymers are addressed, and uncertainty-based active learning is occasionally inefficient. Based on the dependency on the material descriptors, active learning tends to produce a better black-box functions than random sampling when the dimensions of the descriptor are small. The results indicate that active learning is occasionally inefficient in obtaining a better black-box function in materials science.

**Supplementary Information:**

The online version contains supplementary material available at 10.1038/s41598-024-76800-4.

## Introduction

In materials science, materials are synthesized based on the information required to develop these (such as compositions, structures, and processes), and their properties are measured. It can be considered as a function of the information on materials as inputs and material properties as outputs. This function is called a black-box function (BBF) because it cannot be described analytically. To approximate a BBF in materials science, machine learning (ML) models can be used to accurately predict material properties for various inputs. Recently, the concept of black-box optimization (BBO) has attracted attention for the efficient optimization of the inputs of BBF^1–3^. In BBO, the potential materials are selected using an ML model approximating the BBF. Their properties are obtained experimentally or by simulations. Using the newly obtained data, the ML model is updated continually through optimization. Although it is well known that the input of a BBF can be explored efficiently by BBO^4–9^, this approach does not ensure the accuracy of the BBF approximation.

The training dataset and the input of a BBF determine the accuracy of the BBF approximation. To obtain a better input, feature selection methods are useful^10–12^. However, in the case where we do not have enough training data, it is difficult to select better input because it may be highly data dependent. To prepare informative training datasets for constructing a fine approximation of a BBF, Active Learning (AL)^13–16^ is useful. AL has been studied in informatics primarily for classification tasks. It addresses the problem of selecting unlabeled data to be observed to improve the prediction accuracy of ML models. Studies using AL in materials science have also been conducted. A study using ML classification models was the phase diagram construction method^17–19^. This method uses the uncertainty sampling approach^20^ as AL. The most uncertain point in the phase diagram is selected as the next candidate for the experiments. This method can actively sample points near the phase boundaries. A detailed phase diagram can be obtained with 20% of the experiments that would have been required for random sampling. Another study that targeted ML regression models was on X-ray magnetic circular dichroism spectroscopy^21^. The study considered the extent to which the number of experiments required to obtain detailed spectra can be reduced. In this technique, a one-dimensional Gaussian process regression (GPR) learns the data measured from a small number of experiments. An experiment to determine the X-ray energy with the largest variance evaluated by GPR was performed. This procedure was iterated. It was reported that a detailed spectrum could be obtained with 20% of the total number of experiments. Tian et al. investigated the efficiency of AL for different material datasets^22,23^. They reported that a fine approximation of a BBF can be realized by AL when the dimension of the inputs is small. In addition, Jose et al. applied various AL methods for regression to superconductivity data and compared their accuracies^24^. The AL methods considered are model-free methods (which select data only from the input information) and model-based methods (which select data according to a trained ML model). They reported that the accuracy depends strongly on the method. Many methods are less accurate than random sampling. Therefore, the effectiveness of AL is not ensured for regression problems in materials science.

**Fig. 1:**
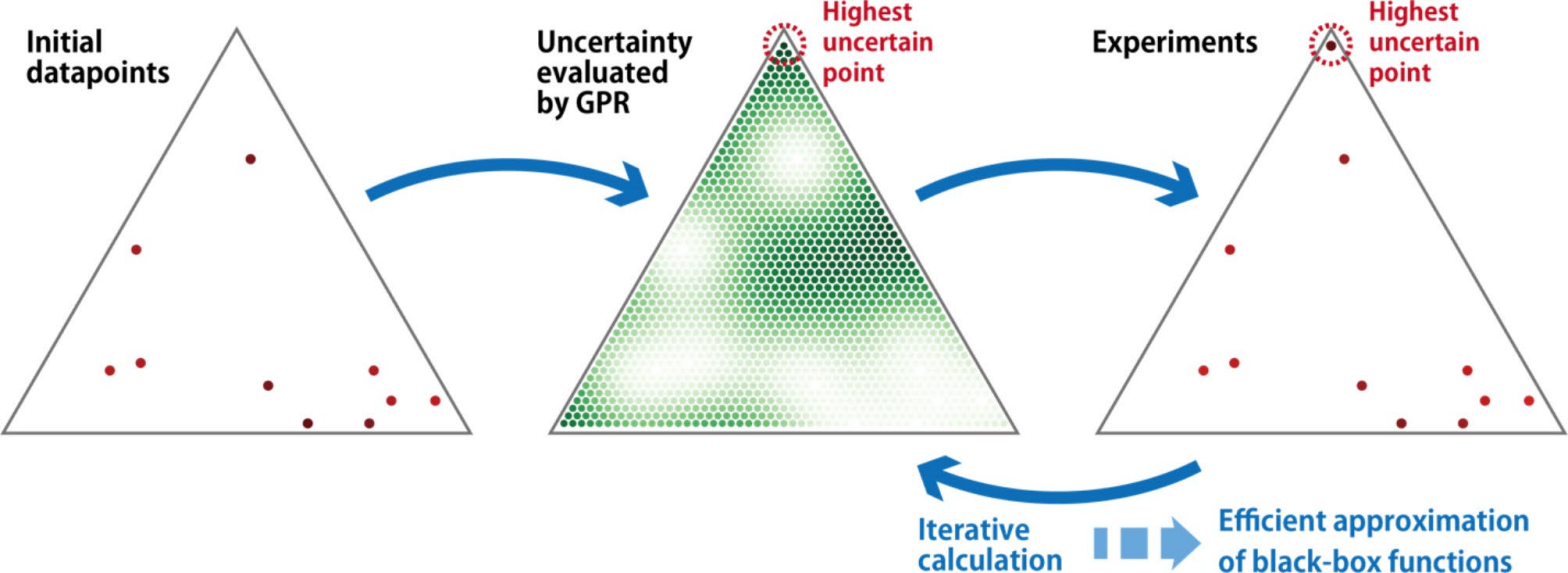
Flow of uncertainty-based active learning. First, a Gaussian process regression (GPR) model is trained by the initial datapoints. Next, the datapoint with the highest uncertainty calculated by GPR is selected, and the property is measured experimentally. By iterating the training of the GPR model, selection of the most uncertain point, and experiments, an efficient approximation of BBFs can be achieved.

In this study, we addressed the performance of AL on various materials datasets when material and molecular descriptors are used. In the fields of materials informatics and chemoinformatics, various descriptors generated by compositions and structures are introduced as input to a BBF. For example, the Matminer descriptors and the Morgan fingerprint are commonly used for inorganic and organic materials, respectively. Their dimensions are often large (45 and 2048 dimensions for the former and the latter, respectively). In addition, to provide useful information for the case where the dataset is built from scratch, we considered the case where the AL starts with almost no data. In such a case, it is difficult to select important elements in the descriptors using feature selection methods due to the small size of the training dataset. Therefore, we focused here on the performance of AL without feature selection for material and molecular descriptors. Furthermore, only model-based AL was considered. Uncertainty sampling is the simplest model-based AL. In uncertainty-based approaches, the most uncertain point defined by the ML prediction results is selected as the next candidate for labeling (Fig. 1). Here, the uncertainty value was calculated using GPR as described in a previous study^21^. The Python package PHYSBO^25^ was used to learn the GPR model. As materials datasets, the liquidus surfaces of ternary systems were prepared as cases in which the inputs for a BBF were given uniformly and defined in a low-dimensional space. Data were generated by CALPHAD calculations with a constant composition step as shown in Fig. 1. Materials databases focus on cases in which the inputs for a BBF are distributed discretely and unbalanced in a high-dimensional feature space. Specifically, datasets of bandgaps and dielectric constants for electrons and lattices calculated by the density functional theory (DFT) for inorganic materials^26^, absorption wavelengths and intensities calculated by DFT for small molecules^27^, and glass transition temperatures of polymers obtained from PoLyInfo^28^ were used. These data have been converted into material or molecular descriptors, and these descriptors are not uniformly distributed in the feature space, i.e. the distribution is unbalanced. We observed that the accuracy of uncertainty-based AL depended strongly on the dataset. In particular, for materials databases, AL tends to produce a finer approximation of a BBF than random sampling when the dimensions of the material descriptors are small.

## Uncertainty-based active learning

This section describes the verification of the performance of the uncertainty-based AL method. Let the dataset $\:\{{\mathbf{x}}_{i},{y}_{i}{\}}_{i=1,\dots\:,N}$ consist of $\:N$ data points with known labels. Here, $\:{\mathbf{x}}_{i}$ is the input for a BBF, and $\:{y}_{i}$ is the output value of the BBF. Here, we focus on the materials datasets, where ${\mathbf{x}}_{i}$ and ${y}_{i}$ are the material descriptors and material properties, respectively. Using known datasets, we hypothetically performed AL and verified its performance according to the following procedure:

1. The validation data, $\:{N}_{\text{v}\text{a}\text{l}}$, are selected in advance from $\:N$ data. To ensure variety in the validation dataset, the interval between the minimum and maximum values of the outputs i.e., $\:[\underset{i}{\text{min}}{y}_{i},\:\underset{i}{\text{max}}{y}_{i}]$, is divided into 100 equal bins. The validation dataset is prepared by randomly selecting a data point from each bin. Therefore, the maximum number of $\:{N}_{\text{v}\text{a}\text{l}}$ is 100.
2. $\:{N}_{\text{i}\text{n}\text{i}}$ data are selected randomly from the remaining $\:{N}_{\text{t}\text{r}\text{a}\text{i}\text{n}}=N-{N}_{\text{v}\text{a}\text{l}}$ data as initial data. This dataset is denoted as$\:\:D=\:\{{\mathbf{x}}_{j},{y}_{j}{\}}_{j=1,\dots\:,{N}_{\text{i}\text{n}\text{i}}}.$
3. Using $\:D$ as training data, the GPR model is learned.
4. Using the GPR model, predictions are made for $\:{N}_{\text{v}\text{a}\text{l}}$, and the prediction accuracy is evaluated.
5. Using the GPR model, predictions are made for the data in $\:{N}_{\text{t}\text{r}\text{a}\text{i}\text{n}}$ that are not included in $\:D$. The data with the largest value of acquisition function was added to $\:D$.
6. Steps 3, 4, and 5 are repeated.

To perform AL, we consider the following four types of acquisition functions used in Step 5:

1 $$\:{f}_{\text{U}\text{S}}\:\left(\mathbf{x}\right)=\sigma\:\left(\mathbf{x}\right),$$

2 $$\:{f}_{\text{T}\text{S}-\mu\:}\:\left(\mathbf{x}\right)=\text{T}\text{S}\left(\mathbf{x}\right)-\mu\:\left(\mathbf{x}\right),$$

3 $$\:{f}_{\text{T}\text{S}}\:\left(\mathbf{x}\right)=\text{T}\text{S}\left(\mathbf{x}\right),$$

4 $$\:{f}_{\text{Random}}\:\left(\mathbf{x}\right)=\text{Uniform}\:\text{random}\:\text{number}\:\text{in}\:\left[\text{0,1}\right],$$

where $\:\mu\:\left(\mathbf{x}\right)$ and $\:\sigma\:\left(\mathbf{x}\right)$ are the mean and standard deviation of the prediction values from the GPR model when $\:\mathbf{x}$ is inputted as a material descriptor. $\:\text{T}\text{S}\left(\mathbf{x}\right)$ is the acquisition function by Thompson sampling. Here, the sampling is performed from the normal distribution with mean $\:\mu\:\left(\mathbf{x}\right)$ and standard deviation $\:\sigma\:\left(\mathbf{x}\right)$, which is obtained by PHYSBO. $\:{f}_{\text{U}\text{S}}\:\left(\mathbf{x}\right)$ is the simplest function for uncertainty-based AL. It selects the point at which the prediction uncertainty is the highest. $\:{f}_{\text{T}\text{S}-\mu\:}\:\left(\mathbf{x}\right)$ is also a function for uncertainty-based AL. It is based on Thompson sampling, which is generated from the normal distribution with mean zero and standard deviation $\:\sigma\:\left(\mathbf{x}\right)$. In contrast, $\:{f}_{\text{T}\text{S}}\:\left(\mathbf{x}\right)$ is an acquisition function for Bayesian optimization used to search for materials with better properties. $\:{f}_{\text{Random}}\:\left(\mathbf{x}\right)$ is the case wherein data are selected randomly in each iteration. It is the basis for determining whether the uncertainty-based AL is effective. The methods using $\:{f}_{\text{US}}\:\left(\mathbf{x}\right)$, $\:{f}_{\text{TS}-\mu\:}\:\left(\mathbf{x}\right)$, $\:{f}_{\text{TS}}\:\left(\mathbf{x}\right)$, and $\:{f}_{\text{Random}}\:\left(\mathbf{x}\right)$ are called US, TS-µ, TS, and Random, respectively.

To evaluate the prediction accuracy in step 4, two cases were considered. That is, GPR or random forest regression (RFR) models were used to predict on the validation dataset. In the first case, the same ML model was used to evaluate the uncertainty and prediction accuracy. Meanwhile, the GPR and RFR models were used to evaluate the uncertainty and prediction accuracy, respectively, in the second case. The prediction accuracy was calculated as the coefficient of determination $\:{R}^{2}$between the true and predicted values for the validation data. PHYSBO was used to learn the GPR models, and scikit-learn^29^ was used to learn the RFR models. To obtain the statistics, 200 independent trials were performed with different initial data selections in Step 2, and the means and standard deviations were calculated.  

## Results

In Section “Liquidus surfaces of ternary alloys”, the liquidus surfaces of the ternary systems are focused on as cases where the inputs are given uniformly and defined in a relatively low-dimensional space. For cases where the inputs were distributed discretely and unbalanced in a high-dimensional feature space, datasets extracted from materials databases of inorganic materials (Section “Inorganic materials datasets”), small molecules (Section “Small molecule datasets”), and polymers (Section “Polymers dataset”) were considered.

### Liquidus surfaces of ternary alloys

**Fig. 2:**
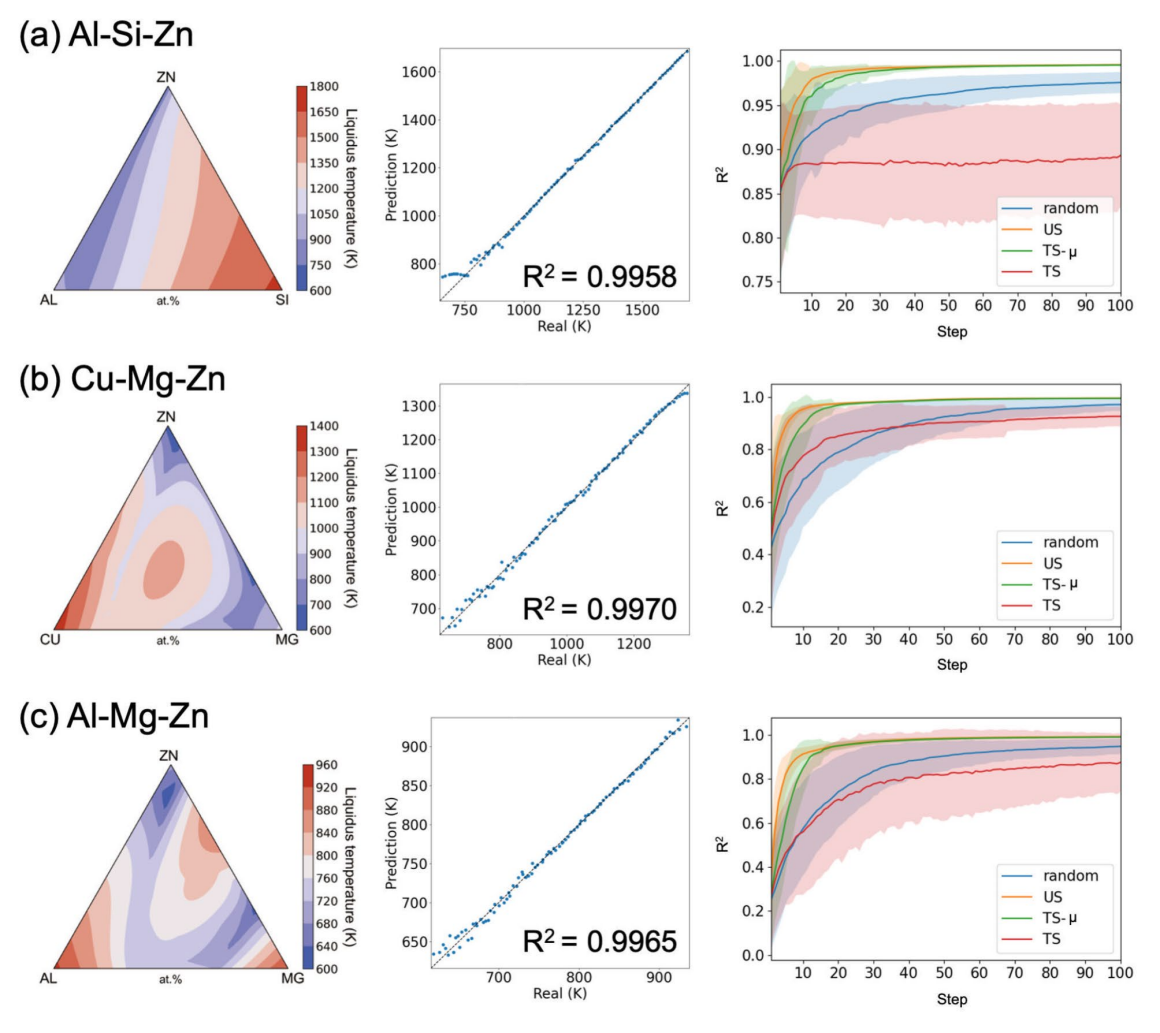
Liquidus temperature (left panels), scatter plot when predicting $\:{N}_{\text{v}\text{a}\text{l}}$ data using all the remaining $\:{N-N}_{\text{v}\text{a}\text{l}}$ data for training (center panels), and the prediction accuracy depending on the iteration steps (right panels) for the (**a**) Al-Si-Zn, (**b**) Cu-Mg-Zn, and (**c**) Al-Mg-Zn systems when the prediction model is GPR. The number of initial data is fixed as $\:{N}_{\text{i}\text{n}\text{i}}\:=\:10$. The 200 independent runs are performed. The mean and standard deviation are depicted as lines and shaded areas, respectively.

We investigated the performance of AL in predicting the liquidus surfaces of ternary alloys obtained from CALPHAD calculations. In this study, the ternary alloys Al-Si-Zn^30^, Cu-Mg-Zn^31^, and Al-Mg-Zn^32^ were focused on. Although the ternary liquidus temperatures can be projected into a two-dimensional space, for simplicity, we used the composition of the three elements as an input for a BBF. The compositions were discretized in 2% increments. The total number of data was $N = 1326$. Figure 2 shows the liquidus temperatures and scatter plots when predicting $\:{N}_{\text{v}\text{a}\text{l}}$ using all the remaining $\:{N-N}_{\text{v}\text{a}\text{l}}$ data for training by GPR. These demonstrate that the liquidus temperatures can be predicted accurately. The prediction accuracies depending on the iteration steps when AL with $\:{N}_{\text{i}\text{n}\text{i}}\:=\:10$ was performed are shown in Fig. 2. Here, GPR was used as the prediction model. In all the cases, US achieved the highest accuracy. That is, better $\:{R}^{2}$ values were obtained when the number of iteration steps was small. Furthermore, US and TS-µ showed similar and better results. However, in all the cases, TS showed the most inferior results. This indicates that the prediction accuracy occasionally does not improve when BBO is performed. Similar results were obtained when the RFR was used as the prediction model (Fig. S1). Thus, we conclude that for the liquidus surface data, the AL is effective in obtaining better BBFs. This conclusion is consistent with the results of a previous study^22^.

### Inorganic materials datasets

In this section, we focus on the physical properties of inorganic materials. We extracted $\:N=1255$ semiconductors from the semiconductor dataset reported in Ref.^26^ containing bandgap values and dielectric constants for electrons and lattices for oxides calculated by DFT. Six descriptors were used to train the ML prediction model based on these properties. Compositional descriptors were obtained from the composition information and properties of the pure elements. Matminer descriptors^33^, magpie descriptors^34^, and Deml descriptors^35^were adopted in this study. As the structural descriptor, orbital field matrix^36^, JarvisCFID descriptors^37^, and radial distribution function were used. These descriptors were generated for $\:N=\text{1255}$ oxides using the matminer python package^33^. The components in the descriptors where all the oxides had an equal value were removed. The dimensions of the descriptors are summarized in Table S1.

**Fig. 3:**
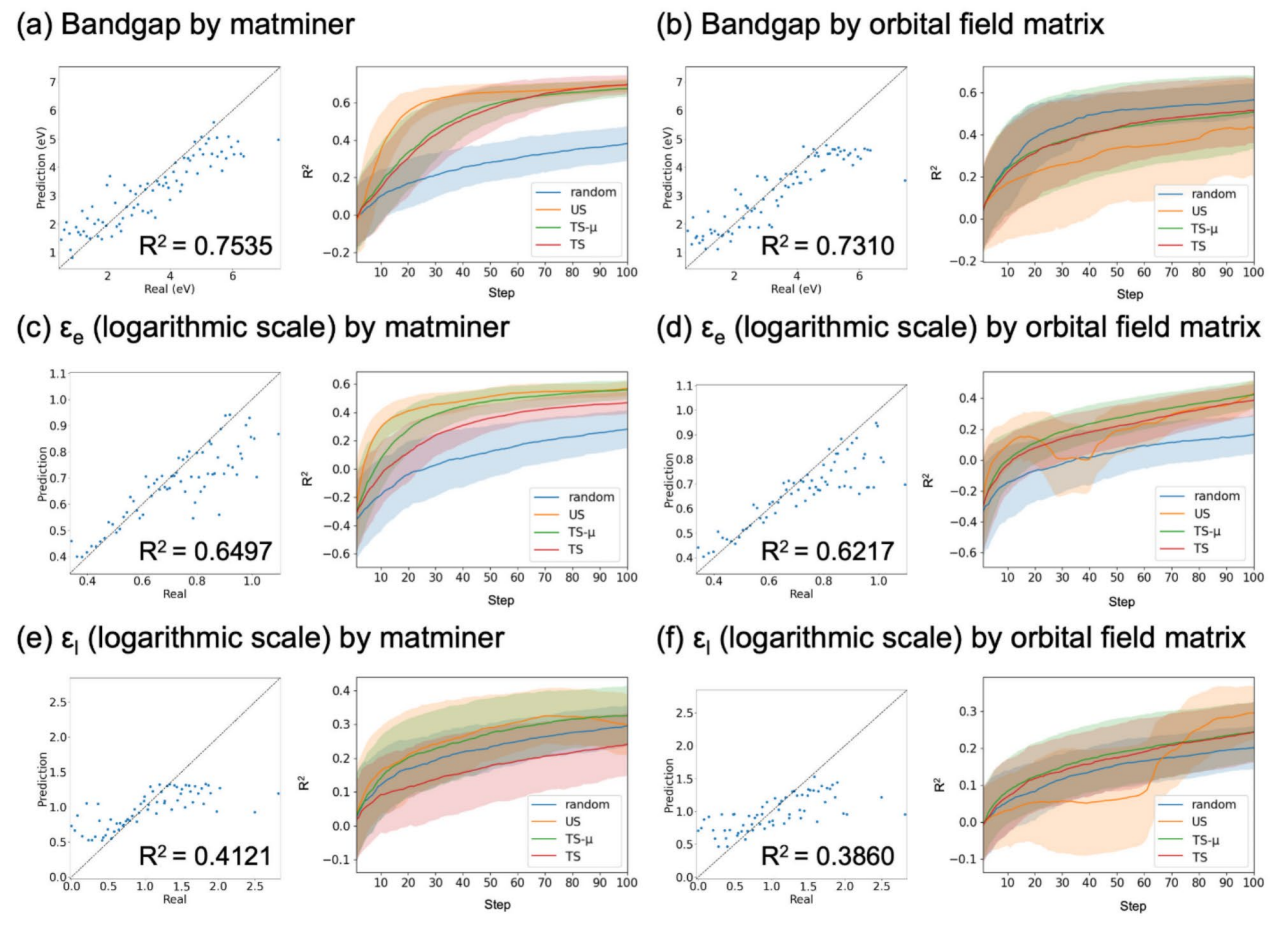
Scatter plots when predicting $\:{N}_{\text{v}\text{a}\text{l}}$ data using all the remaining $\:{N-N}_{\text{v}\text{a}\text{l}}$ data for training (left panels) and the prediction accuracy depending on the iteration steps (right panels) for bandgaps, dielectric constants for electron $({\varepsilon}_{\text{e}})$ and lattice $({\varepsilon}_{\text{l}})$ by matminer and orbital field matrix. The ML model is trained by GPR. The 200 independent runs are performed, and the mean and standard deviation are plotted as lines and shaded areas, respectively.

Figure 3 shows the scatter plots when predicting $\:{N}_{\text{v}\text{a}\text{l}}$ using all the remaining $\:{N-N}_{\text{v}\text{a}\text{l}}$ data for training when the prediction model was GPR. The results by matminer descriptors and orbital field matrix are shown here. The other results are summarized in Figs. S2 and S3. For the dielectric constants, a logarithmic scale was adopted because its prediction accuracy is better than that of the normal scale. The results showed that it is difficult to predict the dielectric constant of a lattice using both compositional and structural descriptors. To validate the AL strategy, the prediction accuracy depending on the number of iteration steps when $\:{N}_{\text{i}\text{n}\text{i}}\:=\:10$ is shown in Fig. 3. When the matminer descriptor was used, US and TS-µ performed better than random sampling for the three physical properties. However, when the orbital field matrix was used, the AL strategy was occasionally ineffective. Thus, for an inorganic dataset, the performance of AL depends strongly on the material properties and descriptors. Similar results were obtained when an RFR model was used to evaluate the prediction accuracy (Figs. S4 and S5).

### Small molecule datasets

**Fig. 4:**
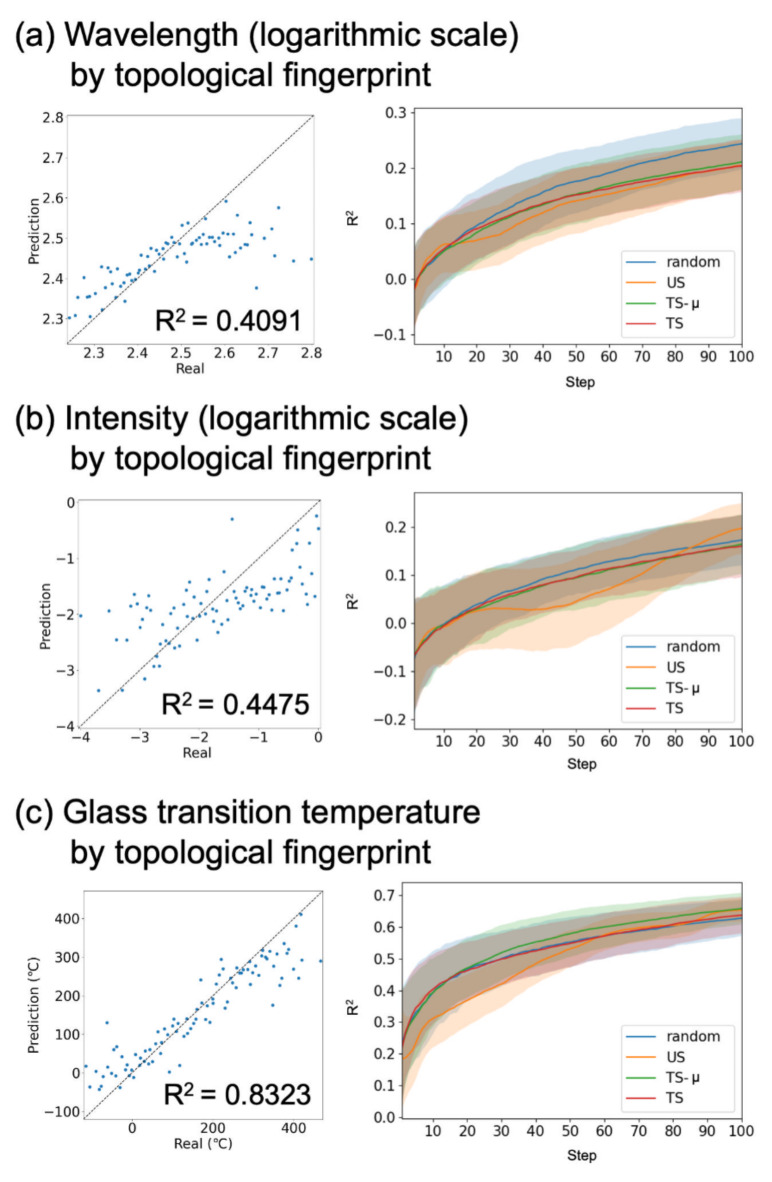
Scatter plots when predicting $\:{N}_{\text{v}\text{a}\text{l}}$ data using all the remaining $\:{N-N}_{\text{v}\text{a}\text{l}}$ data for training (left panels) and the prediction accuracy depending on the iteration steps (right panels) for (**a**) the absorption wavelength prediction, (**b**) intensity prediction in small molecule dataset, and (**c**) the glass transition temperature prediction in homopolymer dataset. The ML model is trained by GPR. The 200 independent runs are performed, and the mean and standard deviation are depicted as lines and shaded areas, respectively.

Next, we considered the performance of AL for the calculated absorption wavelengths and intensities of the small molecules. We extracted $N=1255$ molecules from the ZINC database^38^ for our analyses to arrange the amount of data used in Sections  “Inorganic materials datasets” and “Polymers dataset”. Their properties were calculated using the DFT in Ref.^27^. SMILES notation is used to describe the structure of the molecules. These strings need to be converted into numerical vectors to train an ML prediction model. Here, the Morgan fingerprint^39^, MACCS key, and topological fingerprint obtained using RDKit^40^ were used. The dimensions of each descriptor are summarized in Table S1. Figure 4(a) and (b) show the scatter plots when predicting the logarithmic scale of each property for $\:{N}_{\text{v}\text{a}\text{l}}$ data using all the remaining $\:{N-N}_{\text{v}\text{a}\text{l}}$ data by GPR. For these figures, a topological fingerprint was used. The other cases are summarized in Fig. S6. Figure 4(a) and (b) show the prediction accuracy depending on the iteration steps when AL with $\:{N}_{\text{i}\text{n}\text{i}}\:=\:10$ was performed. No difference in prediction accuracy between the random sampling and AL methods was observed in any case. When an RFR model was used, all acquisition functions gave similar results (Fig. S7). Thus, for our small-molecule datasets, where wavelength and intensity are focused as material properties, the AL strategy is not effective and random sampling is sufficient to obtain better BBFs.

### Polymers dataset

Finally, uncertainty-based AL was validated using a polymer database. Homopolymers recorded in the PoLyInfo database^41^ were used. Their properties were set to the glass transition temperature. From the PoLyInfo database, we extracted $\:N=1255$ data points for our analyses to arrange the number of data used in Sections “Inorganic materials datasets” and “Small molecule datasets”. In the PoLyInfo database, the polymers are represented by their monomer structures using the SMILES format. The SMILES is converted into Morgan fingerprints, MACCS key, and topological fingerprint by RDKit. The dimensions of each descriptor are summarized in Table S1. The results obtained when topological fingerprints were used are summarized in Fig. 4. The other cases are summarized in Fig. S8. The figure shows that the glass transition temperature of $\:{N}_{\text{v}\text{a}\text{l}}$ data can be predicted accurately using all the remaining $\:{N-N}_{\text{v}\text{a}\text{l}}$ data. However, there was no difference in prediction accuracy between the random sampling and AL methods. In the first half of iterations, the results obtained by US were inferior to those obtained by random sampling. Regardless of whether we used other descriptors or the RFR, the AL strategy was not more effective than random sampling (Fig. S9). For the material datasets considered in Sections “Inorganic materials datasets”, “Small molecule datasets”, and “Polymers dataset”, even when BBO was performed (i.e., TS was used as an acquisition function), the accuracy of approximating a BBF was similar to that for random sampling.

## Discussion

### Relationship between data distribution and performance of AL

**Fig. 5:**
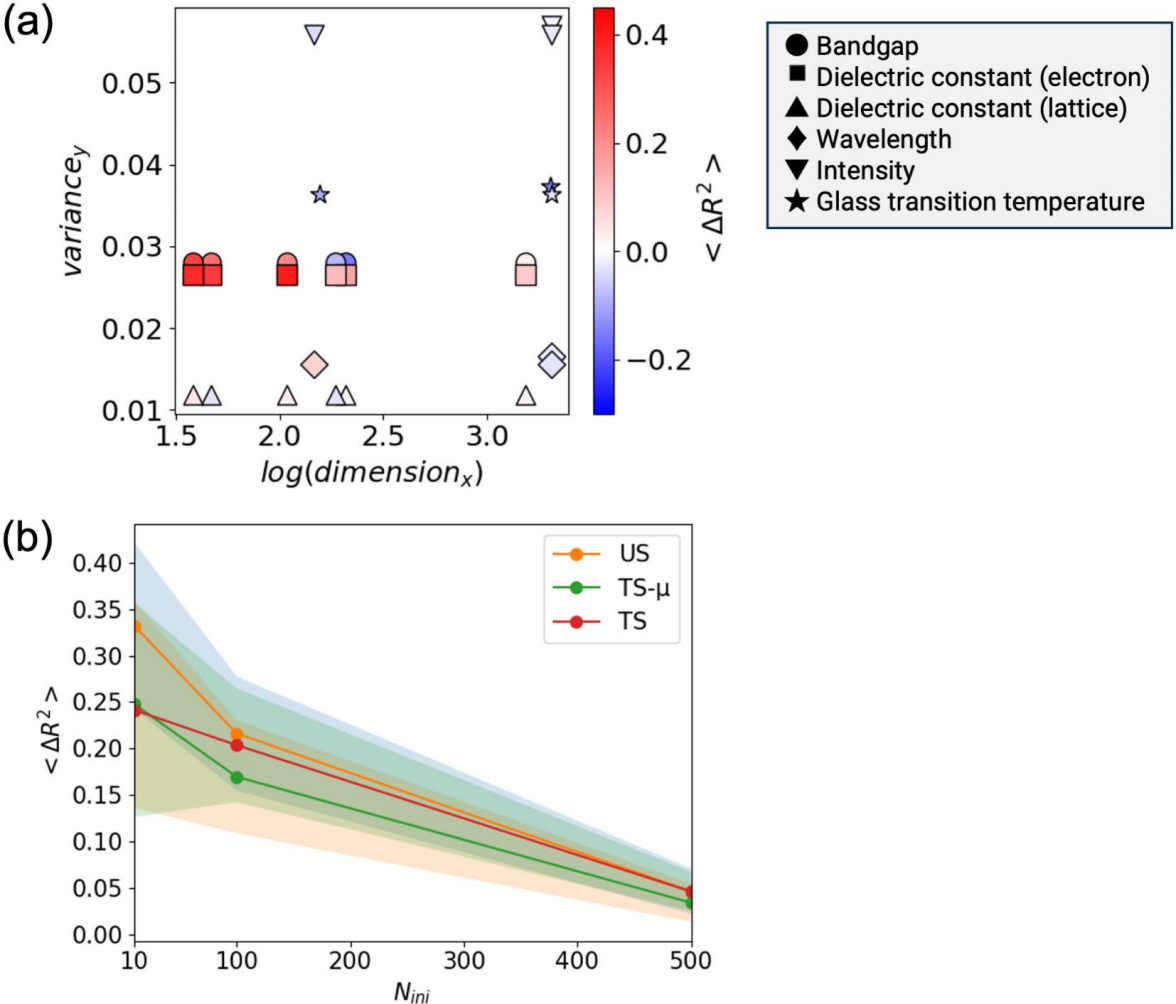
(**a**) Results of $\langle{\Delta}{R}^{2}\rangle$ in a two-dimensional space, where the variance of the objective functions $\:\{{y}_{i}{\}}_{i=1,\dots\:,N}$ is vertical axis and the dimension of the inputs for a BBF is horizontal axis, when $\:{N}_{\text{i}\text{n}\text{i}}\:=\:10$. (**b**) Results of $\langle{\Delta}{R}^{2}\rangle$ for these properties after 100 iterations when $\:{N}_{\text{i}\text{n}\text{i}}\:=\:10,\:100$, and $500$ for the bandgap with the matminer descriptor. The ML model is trained by GPR. The depicted dimensions in Panel (**a**) are active dimension which is summarized in Table S1.

The following equation introduces an indicator of whether AL performs better than random sampling:

5 $$\langle{\Delta}{R}^{2}\rangle=\frac{1}{{N}_{\text{s}\text{t}\text{e}\text{p}}}{\sum\:}_{i=1}^{{N}_{\text{s}\text{t}\text{e}\text{p}}}\left({R}_{i,\text{A}\text{L}}^{2}-{R}_{i,\text{r}\text{a}\text{n}\text{d}\text{o}\text{m}}^{2}\right),$$

where $\:{R}_{i,\text{A}\text{L}}^{2}$ and $\:{R}_{i,\text{r}\text{a}\text{n}\text{d}\text{o}\text{m}}^{2}$ are $\:{R}^{2}$ values between the real and predicted values for $\:{N}_{\text{v}\text{a}\text{l}}$ data at the $\:i$th step when AL and random sampling are performed, respectively. Here, $\:{N}_{\text{s}\text{t}\text{e}\text{p}}$ was set to 100 steps, and US was used as the acquisition function for AL. $\langle{\Delta}{R}^{2}\rangle>0$ implies that AL produced a better prediction model than random sampling. $\langle{\Delta}{R}^{2}\rangle<0$ implies that AL was not effective. Figure 5(a) shows the results of $\langle{\Delta}{R}^{2}\rangle$ on a two-dimensional space, where the variance value of the output values $\:\{{y}_{i}{\}}_{i=1,\dots\:,N}$ and the dimension of the descriptors are used as each axis, when $\:{N}_{\text{i}\text{n}\text{i}}\:=\:10$. To evaluate the variance, the output values were normalized such that the minimum and maximum values were zero and one, respectively. The prediction model was GPR. The results for the inorganic materials, small molecules, and polymer datasets were summarized. Figure 5(a) shows that AL tends to produce a better prediction model than random sampling when the descriptor dimension is small. In general, it is well known that the performance of Bayesian optimization degrades for higher dimensions^42^. Various methods such as REMBO^43^, LINEBO^44^, and SAASBO^45^ have been developed to address this problem. In approximating the BBF, the performance may also be improved by considering the process of addressing the high dimensions applied in the above studies. However, no relationship was observed between the variance of the output values and the effectiveness of AL. A similar trend was observed when the RFR was used as the prediction method (see Fig. S10). In addition, we investigated the data distributions using principal component analysis implemented in scikit-learn^29^, and results are summarized in Figs. S11 and S12. However, clear relationship between distributions and performance of AL cannot be extracted.

### Relationship between the number of initial data and performance of AL

We considered the efficiency of AL and random sampling depending on the number of initial data points. Using a matminer descriptor for the bandgap in inorganic materials, it was verified that AL performed better than random sampling for $\:{N}_{\text{i}\text{n}\text{i}}\:=\:10$ (see Fig. 3). Figure 5(b) shows the result of $\langle{\Delta}{R}^{2}\rangle$ for the bandgap after $\:{N}_{\text{s}\text{t}\text{e}\text{p}}\:=\:100$ iterations when $\:{N}_{\text{i}\text{n}\text{i}}\:=\:10,\:100$, and $500$. As $\:{N}_{\text{i}\text{n}\text{i}}$ increased, $\langle{\Delta}{R}^{2}\rangle$ approached zero. This indicated that the performance of AL is approximately equal to that of random sampling when $\:{N}_{\text{i}\text{n}\text{i}}$ is sufficiently large. This implies that if $\:{N}_{\text{i}\text{n}\text{i}}$ is sufficiently large, a better prediction model can be constructed in the initial stage. From this point onward, adding more data did not significantly alter the prediction accuracy, and the difference between AL and random sampling was insignificant.

## Conclusion

In this study, we tested the performance of AL in constructing a fine approximation of the BBF on several material datasets. First, we focused on the BBF for the liquidus temperature in the ternary systems, whose inputs were given as continuous values. This continuous space was discretized uniformly. The next point to be evaluated was selected using an uncertainty-based AL approach. The results showed that uncertainty sampling produced a finer approximation of the BBF more efficiently than random sampling. However, when a material database was used in which the inputs were distributed discretely and unbalanced in a high-dimensional space, the results depended strongly on the dataset. In particular, when the dimensions of the descriptors were high, the difference between AL and random sampling was not verified. Using material descriptors, the inputs for a BBF can straightforwardly be over 100-dimensional. Thus, for many material datasets, AL is ineffective at producing a prediction model with a high accuracy compared with random sampling.

## Electronic supplementary material

Below is the link to the electronic supplementary material.

Supplementary Material 1

## Data Availability

The data that support the findings of this study are available from the corresponding author upon reasonable request.
