# Supplementary material for: Performance of uncertainty-based active learning for efficient approximation of black-box functions in materials science
Source: Sci Rep. 2024 Nov 6;14:27019. doi: 10.1038/s41598-024-76800-4 (PMC11541781; doi:10.1038/s41598-024-76800-4)
Supplement: Supplementary file 1 — Supplementary Material 1 [file 41598_2024_76800_MOESM1_ESM.pdf]

Supplementary information for

Performance of uncertainty-based active learning for efficient approximation of black-box functions in materials science

Ai Koizumi[1*], Guillaume Deffrennes[2], Kei Terayama[3,4,5*], and Ryo Tamura[1,5,6*]

1 Center for Basic Research on Materials, National Institute for Materials Science, 1-1 Namiki, Tsukuba, Ibaraki 305-0044, Japan

2 Univ. Grenoble Alpes, CNRS, Grenoble INP, SIMaP, F-38000 Grenoble, France

3 Graduate School of Medical Life Science, Yokohama City University, 1-7-29, Suehiro-cho, Tsurumi-ku, Kanagawa 230-0045, Japan

4 MDX Research Center for Element Strategy, Tokyo Institute of Technology, 4259 Nagatsuta-cho, Midori-ku, Yoko-hama, Kanagawa, 226-8501, Japan.

5 RIKEN Center for Advanced Intelligence Project, 1-4-1, Nihonbashi, Chuo-ku, Tokyo 103-0027, Japan.

6 Graduate School of Frontier Sciences, The University of Tokyo, 5-1-5 Kashiwa-no-ha, Kashiwa, Chiba 277-8561, Japan

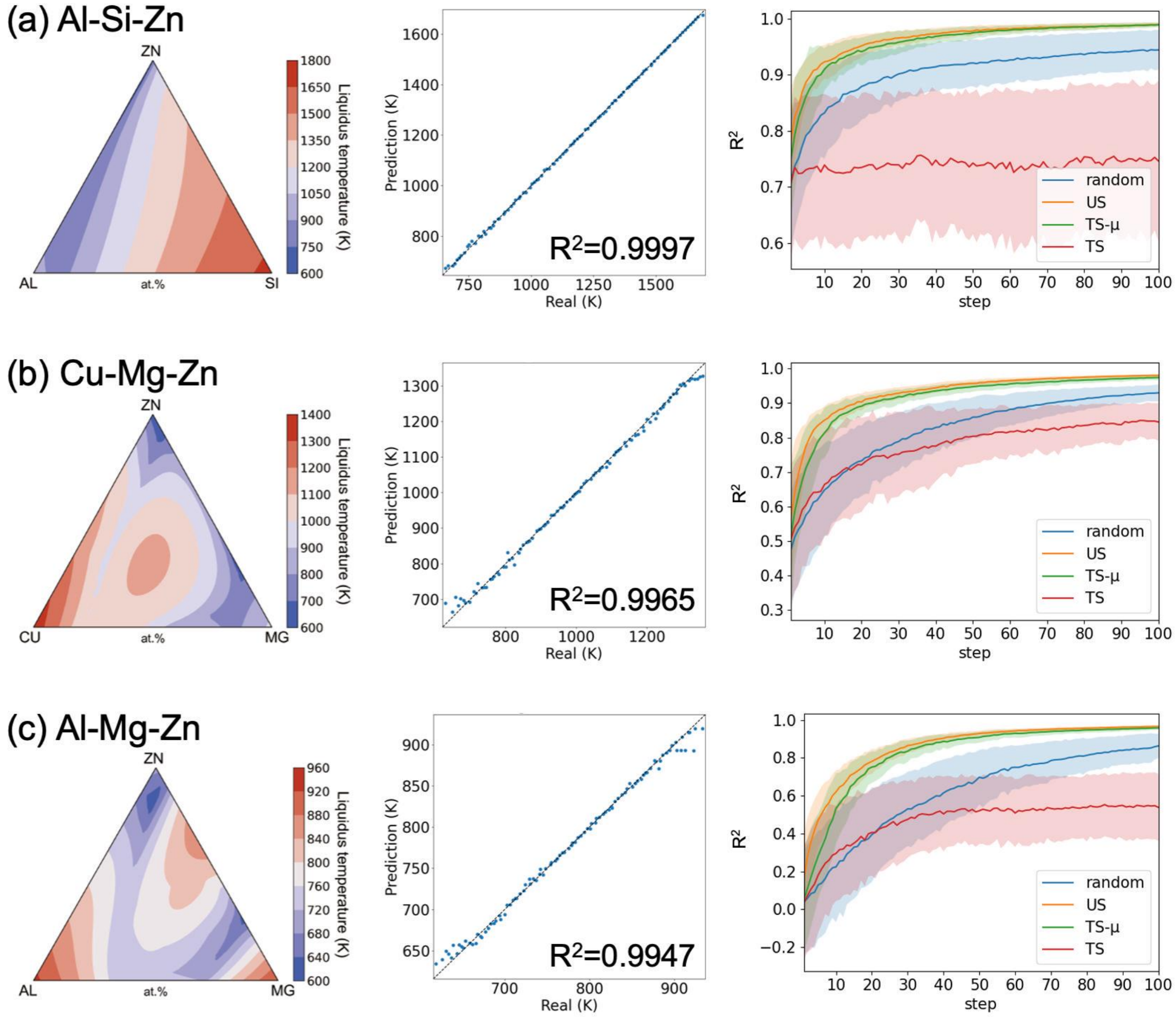

Fig. S1 Liquidus temperature (left panels), scatter plot when predicting N_{val} data using all the remaining $N - N_{\mathrm{val}}$ data for training (center panels), and the prediction accuracy depending on the iteration steps (right panels) for the (a) Al-Si-Zn, (b) Cu-Mg-Zn, and (c) Al-Mg-Zn systems when the prediction model is RFR. The number of initial data is fixed as $N_{\mathrm{ini}} = 10$. The 200 independent runs are performed. The mean and standard deviation are depicted as lines and shaded areas, respectively.

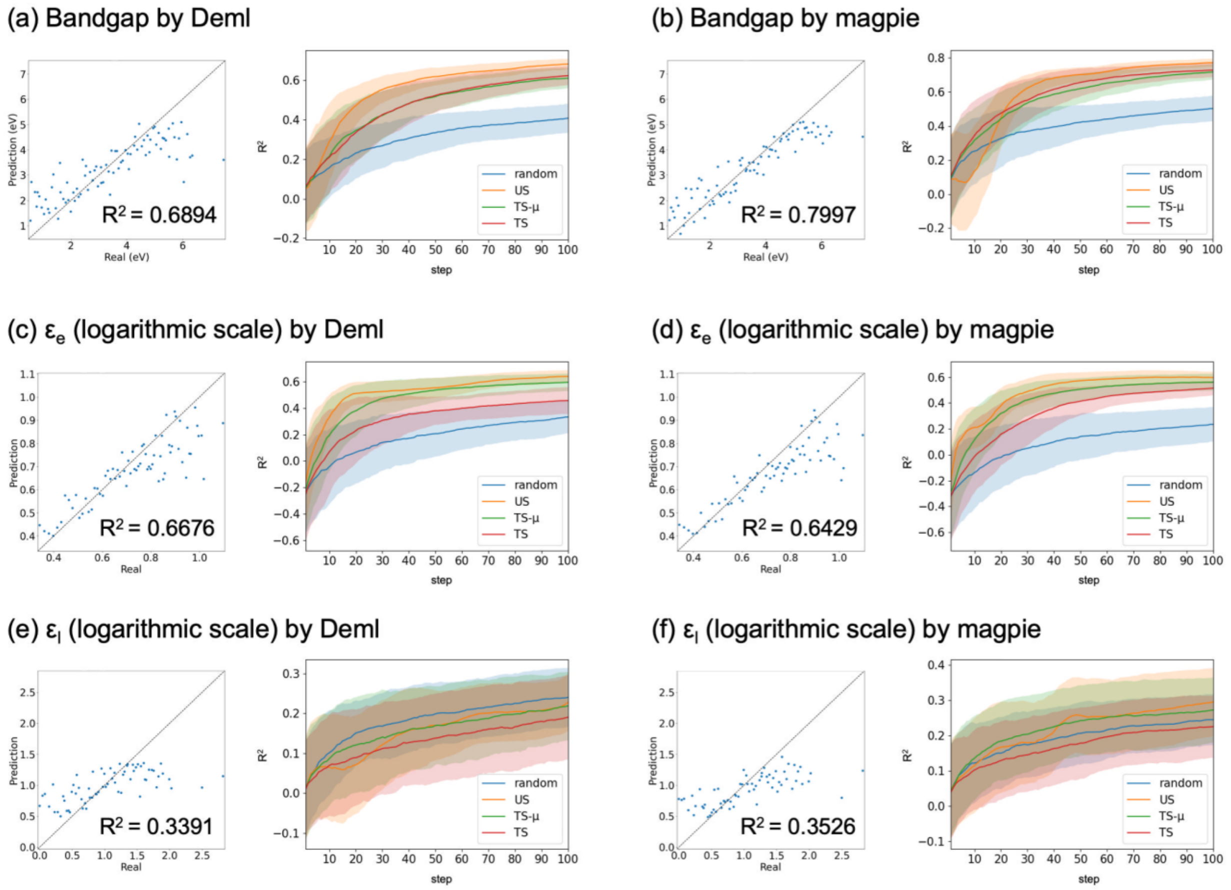

Fig. S2 Scatter plots when predicting N_{val} data using all the remaining $N - N_{\mathrm{val}}$ data for training (left panels) and the prediction accuracy depending on the iteration steps (right panels) for bandgaps, dielectric constants for electron (ε_{e}) and lattice (ε_{l}) by Deml and magpie. The ML model is trained by GPR. The 200 independent runs are performed, and the mean and standard deviation are plotted as lines and shaded areas, respectively.

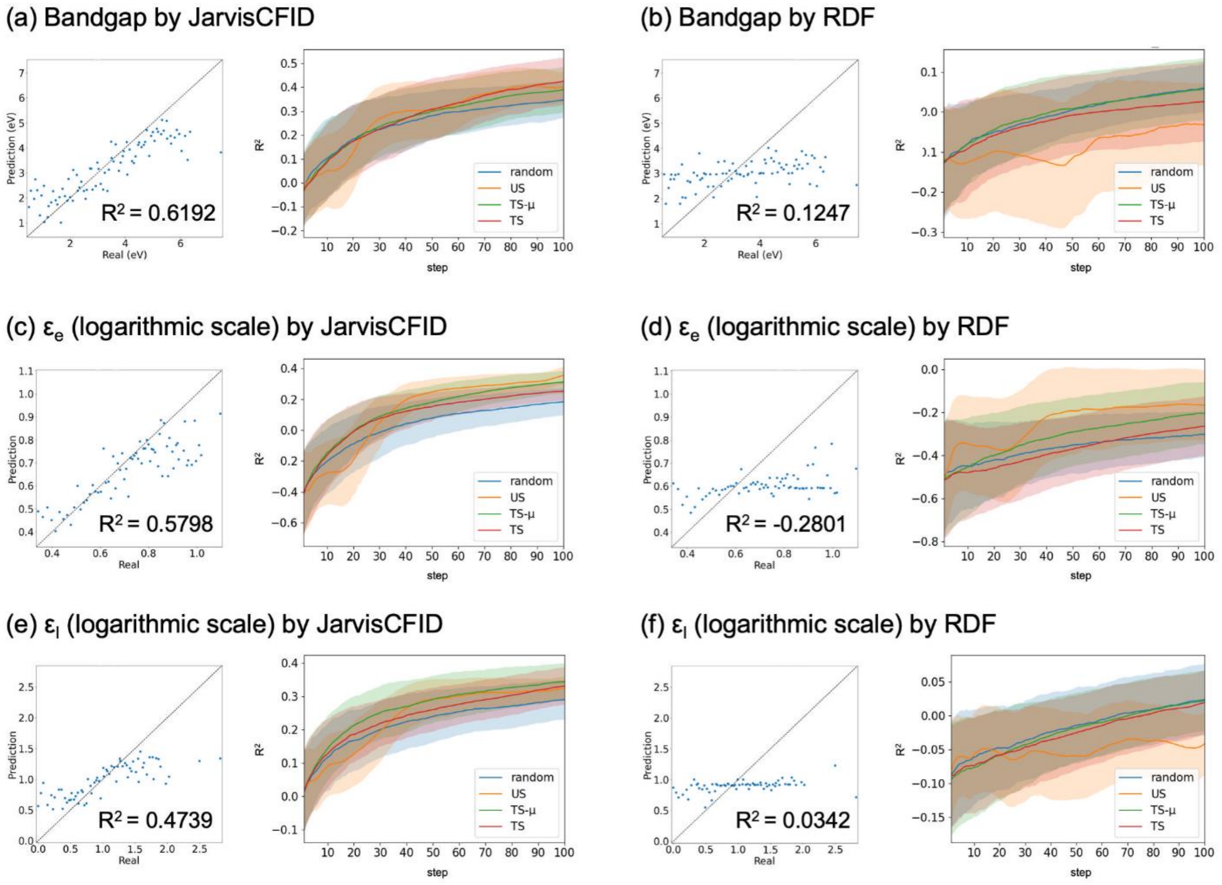

Fig. S3 Scatter plots when predicting N_{val} data using all the remaining $N - N_{\mathrm{val}}$ data for training (left panels) and the prediction accuracy depending on the iteration steps (right panels) for bandgaps, dielectric constants for electron (ε_{e}) and lattice (ε_{l}) by JarvisCFID and radial distribution function (RDF). The ML model is trained by GPR. The 200 independent runs are performed, and the mean and standard deviation are plotted as lines and shaded areas, respectively.

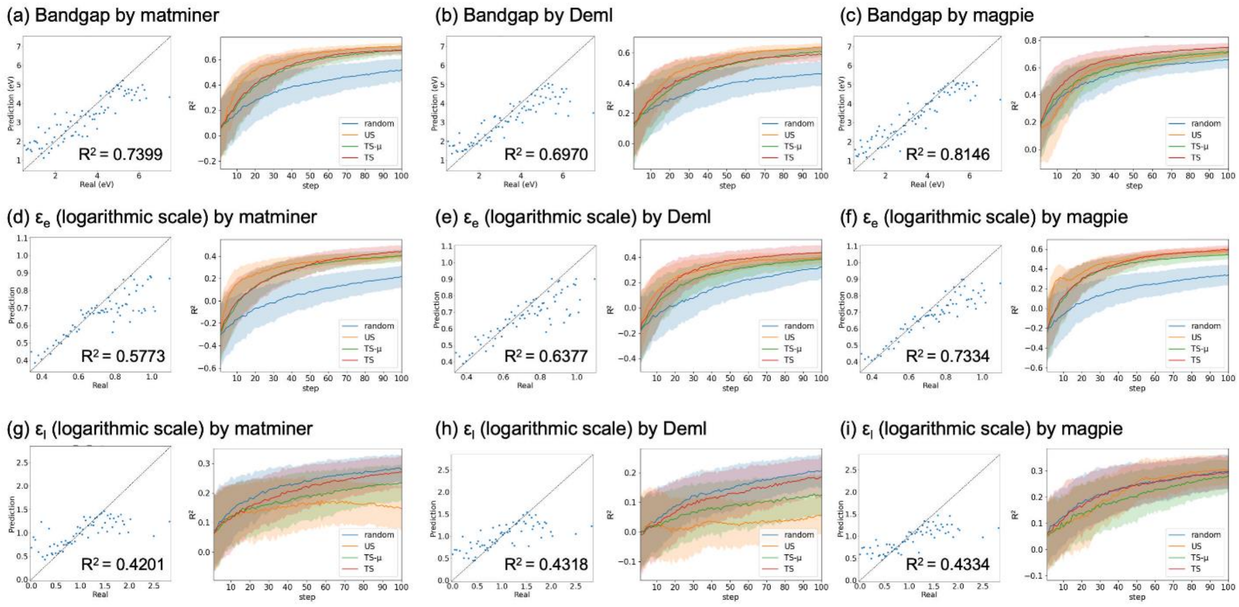

Fig. S4 Scatter plots when predicting N_{val} data using all the remaining $N - N_{\mathrm{val}}$ data for training (left panels) and the prediction accuracy depending on the iteration steps (right panels) for bandgaps, dielectric constants for electron (ε_{e}) and lattice (ε_{l}) by matminer, Deml, and magpie. The ML model is trained by RFR. The 200 independent runs are performed, and the mean and standard deviation are plotted as lines and shaded areas, respectively.

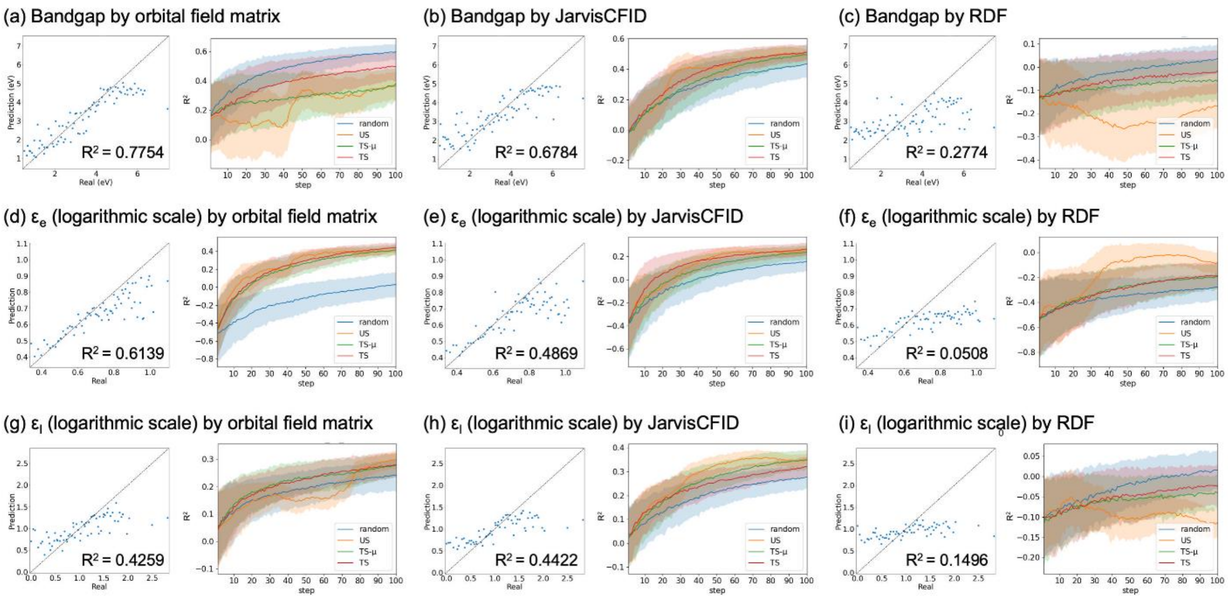

Fig. S5 Scatter plots when predicting N_{val} data using all the remaining $N - N_{\mathrm{val}}$ data for training (left panels) and the prediction accuracy depending on the iteration steps (right panels) for bandgaps, dielectric constants for electron (ε_{e}) and lattice (ε_{l}) by orbital field matrix, JarvisCFID, and radial distribution function (RDF). The ML model is trained by RFR. The 200 independent runs are performed, and the mean and standard deviation are plotted as lines and shaded areas, respectively.

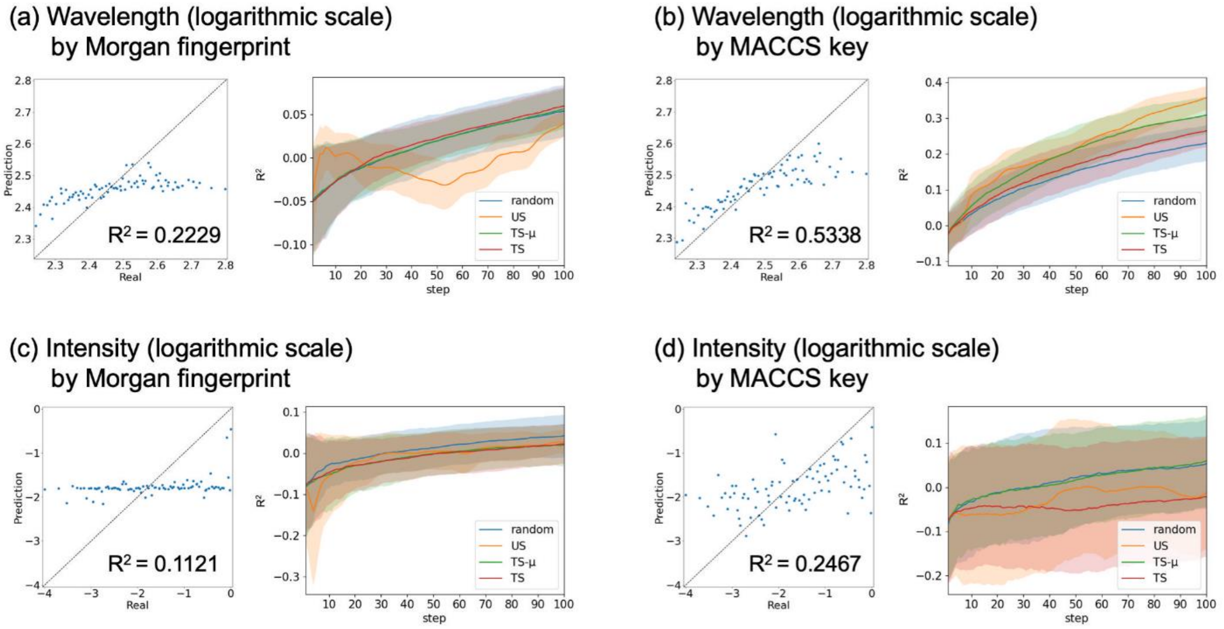

Fig. S6 Scatter plots when predicting N_{val} data using all the remaining $N - N_{\text{val}}$ data for training (left panels) and the prediction accuracy depending on the iteration steps (right panels) for wavelength and intensity by Morgan fingerprint and MACCS key. The ML model is trained by GPR. The 200 independent runs are performed, and the mean and standard deviation are depicted as lines and shaded areas, respectively.

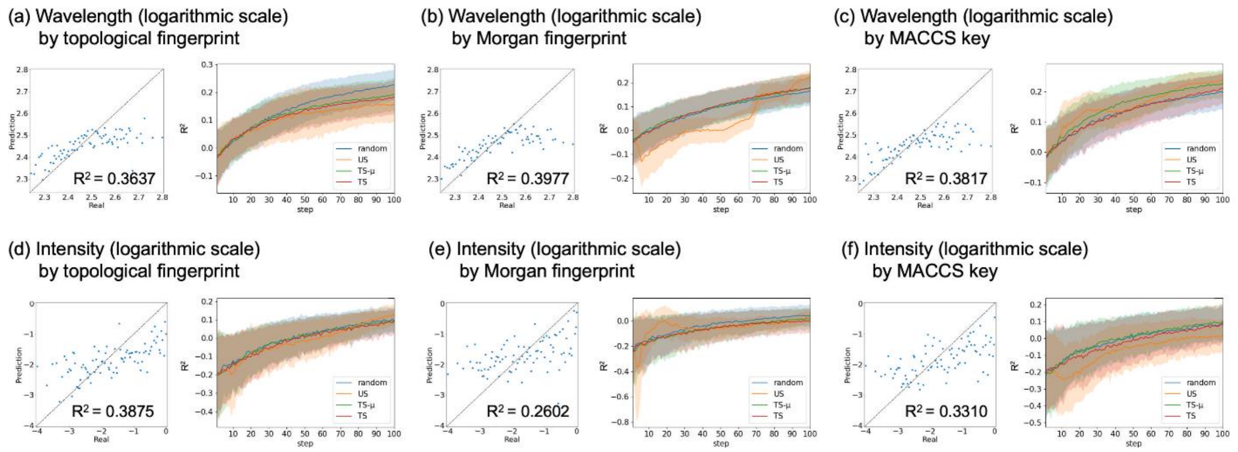

Fig. S7 Scatter plots when predicting N_{val} data using all the remaining $N - N_{\mathrm{val}}$ data for training (left panels) and the prediction accuracy depending on the iteration steps (right panels) for wavelength and intensity by topological fingerprint, Morgan fingerprint, and MACCS key. The ML model is trained by RFR. The 200 independent runs are performed, and the mean and standard deviation are depicted as lines and shaded areas, respectively.

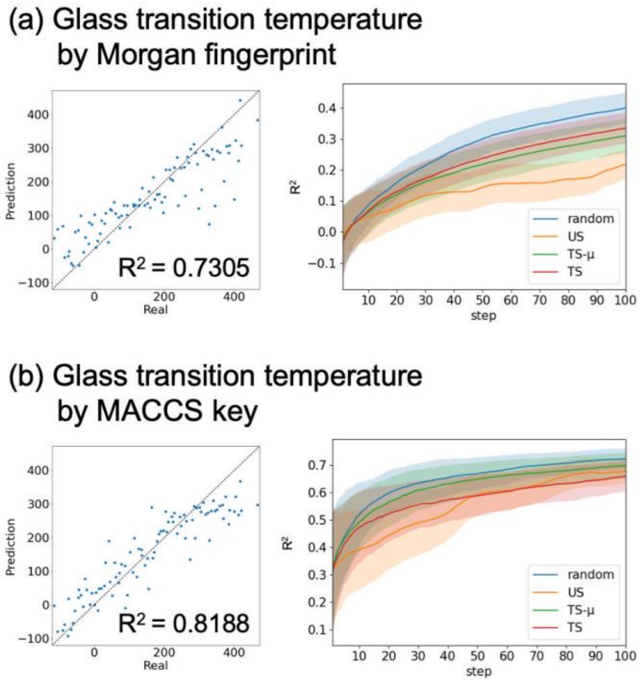

Fig. S8 Scatter plots when predicting N_{val} data using all the remaining $N - N_{\mathrm{val}}$ data for training (left panels) and the prediction accuracy depending on the iteration steps (right panels) for glass transition temperature by Morgan fingerprint and MACCS key. The ML model is trained by GPR. The 200 independent runs are performed, and the mean and standard deviation are depicted as lines and shaded areas, respectively.

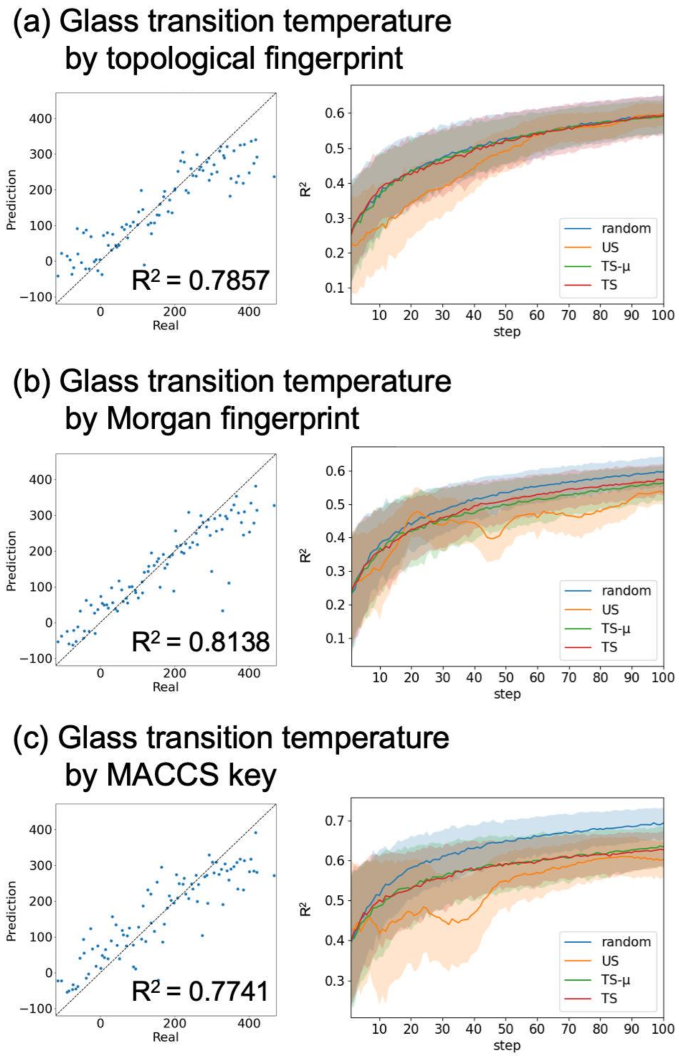

Fig. S9 Scatter plots when predicting N_{val} data using all the remaining $N - N_{\mathrm{val}}$ data for training (left panels) and the prediction accuracy depending on the iteration steps (right panels) for glass transition temperature by topological fingerprint, Morgan fingerprint, and MACCS key. The ML model is trained by RFR. The 200 independent runs are performed, and the mean and standard deviation are depicted as lines and shaded areas, respectively.

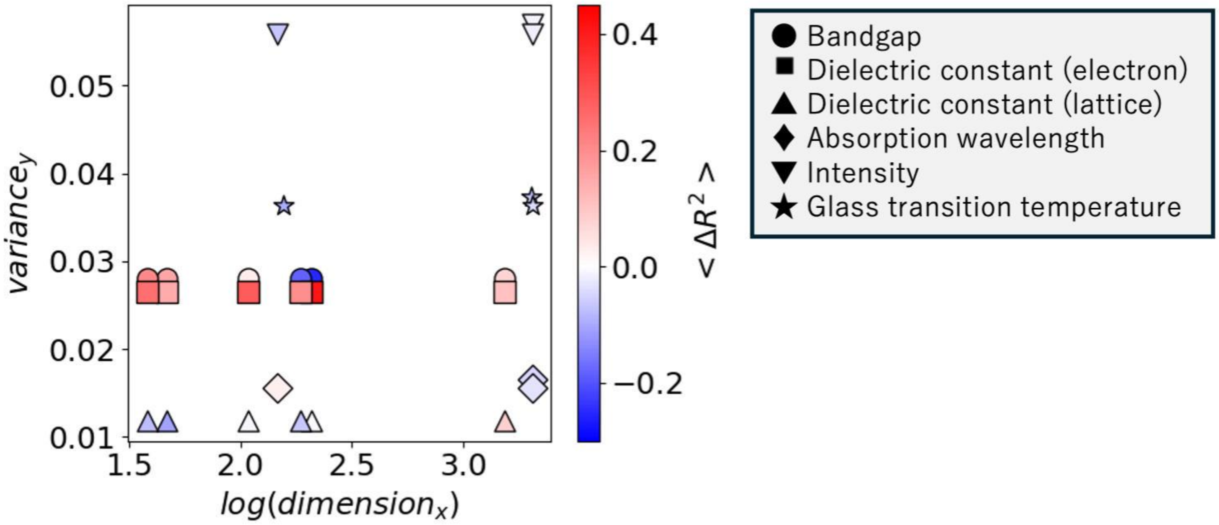

Fig. S10 Results of $\langle \Delta R^2 \rangle$ in a two-dimensional space, where the variance of the objective functions $\{y_i\}_{i=1,\dots,N}$ is vertical axis and the dimension of the inputs for a BBF is horizontal axis, when $N_{\text{ini}} = 10$. The ML model is trained by RFR.

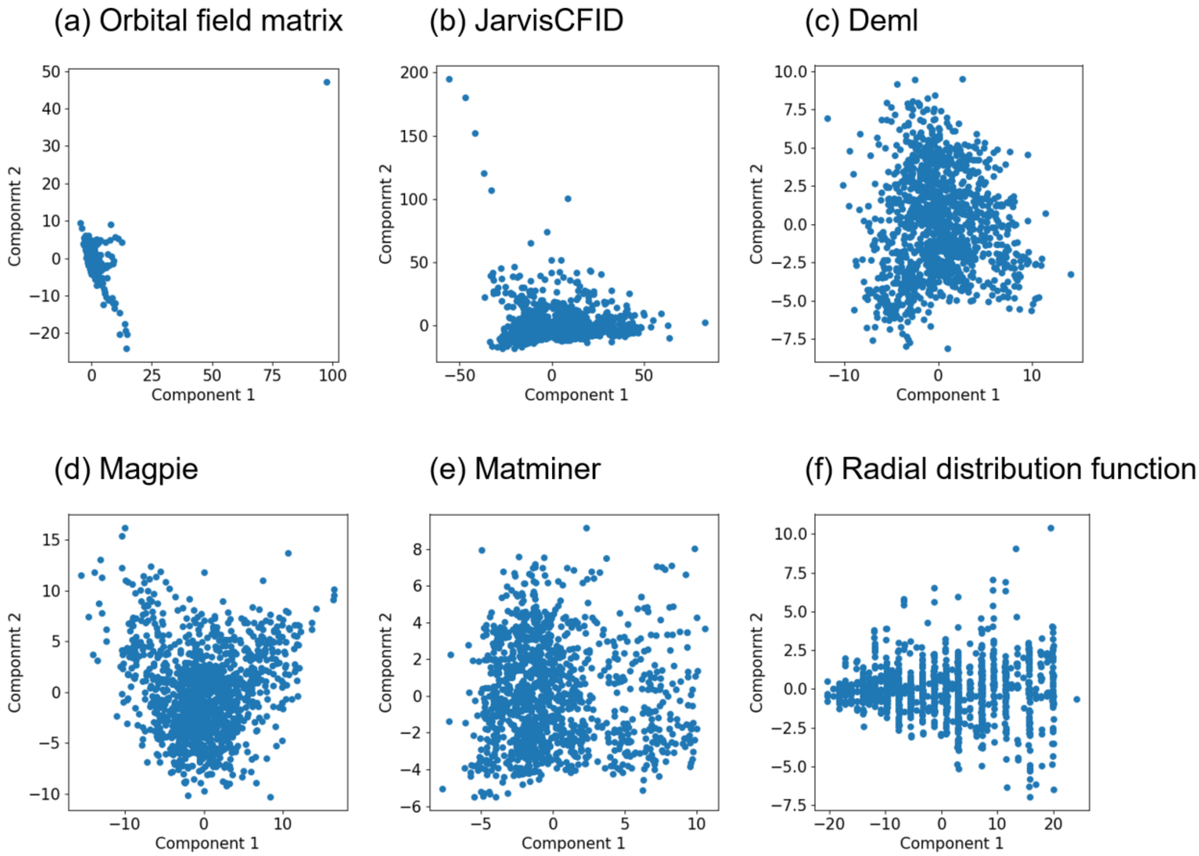

Fig. S11 Results of principal component analysis (PCA) of the descriptors for inorganic materials.

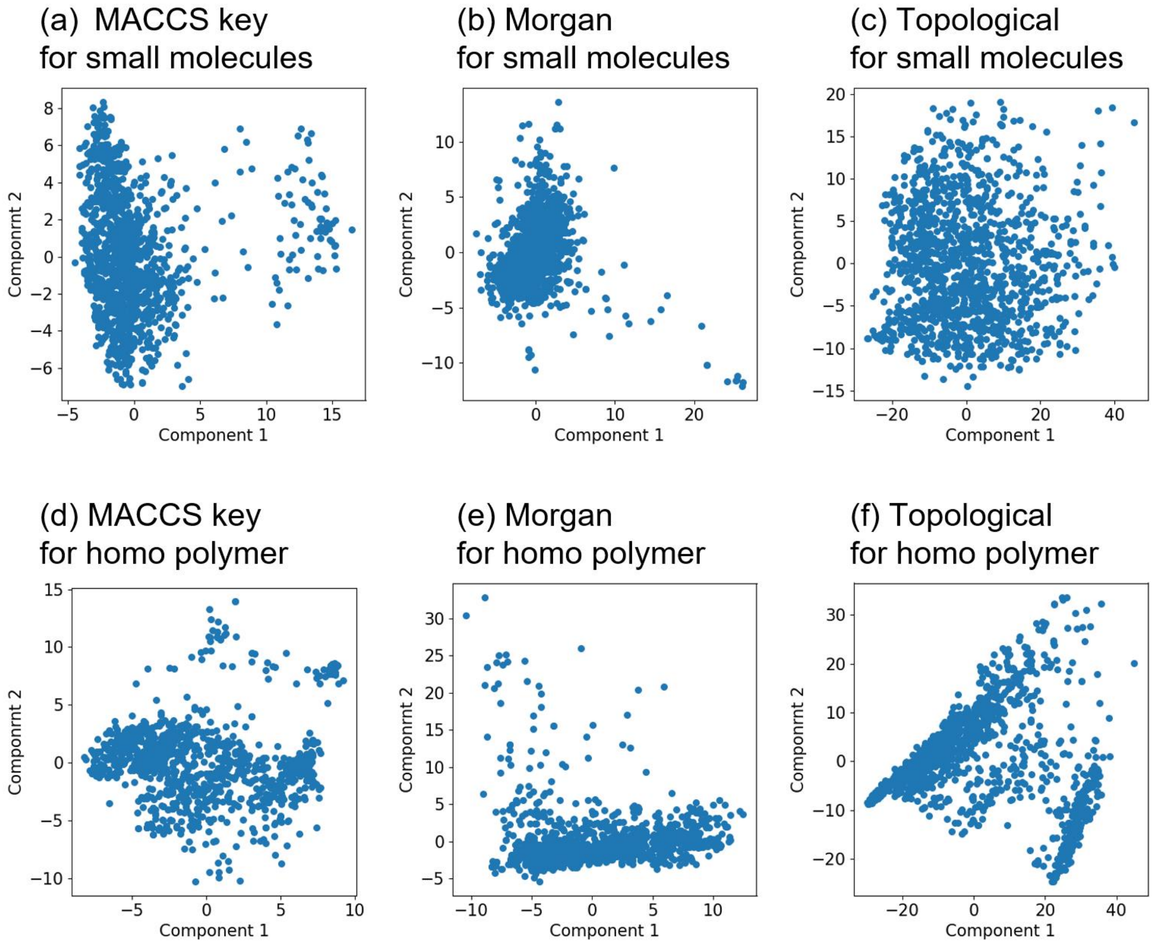

Fig. S12 Results of principal component analysis (PCA) of the descriptors for small molecules and homo polymer.

Table S1. Summary of dimensions of descriptors.

Descriptors	Whole dimension	Active dimension
Orbital field matrix for inorganic materials	1024	208
JarvisCFID for inorganic materials	1557	1537
Deml for inorganic materials	55	47
Magpie for inorganic materials	132	108
Matminer for inorganic materials	45	38
Radial distribution function for inorganic materials	200	187
MACCS key for small molecules	167	146
Morgan fingerprint for small molecules	2048	2048
Topological fingerprint for small molecules	2048	2048
MACCS key for homo polymer	167	157
Morgan fingerprint for homo polymer	2048	2032
Topological fingerprint for homo polymer	2048	2048
